# The relationship between cervical flexor endurance, cervical extensor endurance, VAS, and disability in subjects with neck pain

**DOI:** 10.1186/2045-709X-22-10

**Published:** 2014-03-03

**Authors:** Sergio Parazza, Carla Vanti, Caroline O’Reilly, Jorge Hugo Villafañe, José Miguel Tricás Moreno, Elena Estébanez De Miguel

**Affiliations:** 1 Private practitioner, Modena, Savignano sul Panaro, Italy; 2 School of Physiotherapy, Alma Mater Studiorum, University of Bologna, via Tosarelli 144 40055 Castenaso, Bologna, Italy; 3 Private practitioner, Dublin, Ireland; 4 IRCCS Don Gnocchi Foundation, Milan, Italy; 5 Unidad de Investigación en Fisioterapia, University of Zaragoza, Zaragoza, Spain; 6 Department of Physiatry and Nursing, University of Zaragoza, Zaragoza, Spain

**Keywords:** Neck pain, Physical endurance, Neck muscles, Isometric contraction, Measure

## Abstract

**Background:**

Several tests have been suggested to assess the isometric endurance of the cervical flexor (NFME) and extensors (NEE) muscles. This study proposes to determine whether neck flexors endurance is related to extensor endurance, and whether cervical muscle endurance is related to disability, pain amount and pain stage in subjects with neck pain.

**Methods:**

Thirty subjects (18 women, 12 men, mean **±** SD age: 43 ± 12 years) complaining of neck pain filled out the Visual Analogue Scale (VAS) and the Neck Pain and Disability Scale-Italian version (NPDS-I). They also completed the timed endurance tests for the cervical muscles.

**Results:**

The mean endurance was 246.7 **±** 150 seconds for the NEE test, and 44.9 **±** 25.3 seconds for the NMFE test. A significant correlation was found between the results of these two tests (r = 0.52, p = 0.003). A positive relationship was also found between VAS and NPDS-I (r = 0.549, p = 0.002). The endurance rates were similar for acute/subacute and chronic subjects, whereas males demonstrated significantly higher values compared to females in NFME test.

**Conclusions:**

These findings suggest that neck flexors and extensors endurance are correlated and that the cervical endurance is not significantly altered by the duration of symptoms in subjects with neck pain.

## Introduction

Neck pain (NP) is a condition that is becoming more and more widespread and its associated economic and social costs are ever-increasing [1]. Some possible factors are sedentary work, the increase in activities such as the use of personal computers and Internet, the use of motor vehicles, and changes of work type [2]. Some psychosocial factors are considered as the strongest prognostic factors for NP and contributing factors for slower or less complete recovery [3] and persistent pain [4]. Moreover, these factors influence the perception of pain and disability and decrease the self-efficacy perception [5].

According to various studies, in many cervical conditions such as whiplash associated disorders (WAD) or cervicogenic headache, a dysfunction of deep cervical flexors (DCF) such as longus colli and longus capitis can be found [6,7]. Other studies also describe the deep cervical extensors as being dysfunctional: especially semispinalis, sub-occipital and multifidus muscles [8-10].

From the literature, it seems that muscular dysfunction in the cervical spine refers to changes in structure [8-12] and function [13-15]. Moreover, the following deficiencies have been observed in people affected by NP: reductions in maximal strength, in accuracy of head position during dynamic movements and repositioning, in efficiency of contraction, and in muscle endurance [16].

Recent tests for cervical muscles have been suggested, such as the Cranial Cervical Flexion test [6], which assesses the endurance of the DCF, and the Neck Flexor Muscle test (NFME) [17,18], which evaluates the endurance of both the superficial and the deep cervical flexor muscles. In addition, other tests have recently been proposed to appraise the deep and superficial cervical extensors. The Neck Extensor Endurance test (NEE) [7,19] is probably the most common one, because it can be used in the clinical setting without any complicated tools.

Several studies have shown a relationship between pain and the strength and endurance of flexor muscles [6,19-22], and some research has been done to investigate the relationship between pain and extensor muscles strength [7,19,21]. The relationship between endurance and the time from which the pain was present has been insufficiently studied, showing a lack of correlation between fatigability of some flexor muscles (sternocleidomastoid and anterior scalene) and duration of symptoms in chronic neck pain patients [23]. The purpose of this study was to investigate the isometric endurance of the neck extensor and neck flexor muscles in subjects complaining of acute, subacute or chronic NP, to determine whether there was any relationship between neck flexor muscle endurance, neck extensor muscle endurance, amount of pain and disability, and between endurance and duration of pain.

## Methods

### Subjects

We conducted an observational study. Informed consent was obtained from all subjects and the study was conducted following the ethical rules of the Orthopaedic Manual Physiotherapy Masters Research Committee at the University of Zaragoza (Spain). All procedures were carried out in accordance with the Declaration of Helsinki. Prior to participation in the study, all subjects signed an informed consent form.

Thirty-four subjects, 60% female (mean ± SD age: 43 ± 12 years), with a primary complaint of NP were assessed at the Fisioplus Private Practice (Savignano sul Panaro, Modena, Italy). The recruitment of eligible subjects was carried out by means of notices on the Internet and on information boards located in some general practitioners consulting rooms in the area of the clinic.

To be included in the study, the subjects had to be between 18–80 years of age and have a diagnosis of NP, including a thorough anamnesis and physical examination. The exclusion criteria were: the presence of positive neurological signs (abnormalities on myotomes, dermatomes and reflexes), systemic, muscular, or connective tissue disorders, cognitive disorders, and a history of trauma in the last six months.

### Procedure of the tests and questionnaires

The subjects included in the study completed the Visual Analogue Scale (VAS) and the Neck Pain and Disability Scale - Italian version (NPDS-I) [24]. The score of NPDS-I can range between 0 and 100 [25], where 0–30 it is considered to be low disability; 30–70 is fairly high disability; and 70–100 is considered high disability.

Subjects were asked not only to state their pain intensity, but also to define the location of their pain on a body chart as well as the duration and frequency of their symptoms. The investigator showed pictures to the subjects (taken from studies by Harris et al. [26] and Lee et al. [7])*,* in order to familiarize them with the tests and explained in detail how the tests would be carried out [27]. A fluid inclinometer (G314S Plasty, Milano, Italy) was used to check the degrees of head displacement on the sagittal plane.

### Neck Flexor Muscle Endurance Test (NFME test)

**Figure 1:**
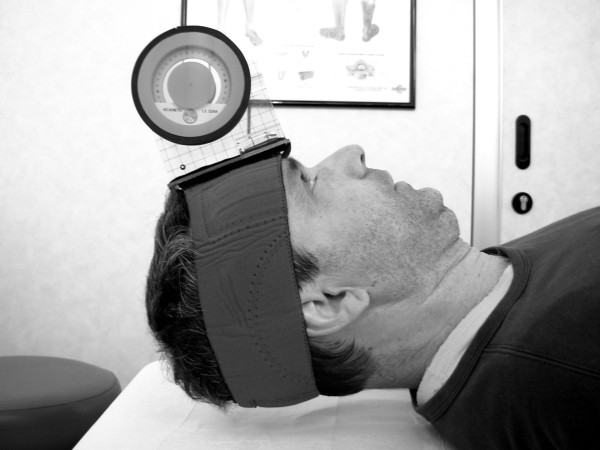
**NFME test.** The subject is lying supine with a fluid inclinometer on his forehead. The position of the subject’s hands, the examiner’s hand underneath the subject’s head, and the line drawn across skin folds are not shown in this picture.

The test was performed in the supine and crook lying positions [26,28,29]. With the chin maximally tucked and maintained isometrically, the subject lifted the head and neck until the head was approximately 2.5 cm off the plinth while maintaining the chin retracted to the chest (Figure 1). During the test verbal commands such as “tuck your chin in” or “hold your head up” were given whenever there was a loss of chin tuck. The test was stopped if the subject’s head touched the investigator’s hand for more than one second, if the skin folds began to separate due to a loss of chin tuck for the same amount of time, or if the subject wanted to stop because of fatigue or pain. The test was also stopped if the subject lost more than 5° for over two seconds (degrees measured with the inclinometer).

Holding time was recorded in seconds with a stopwatch and the reason for stopping the test was noted. Then the subject was asked to sit up and the Velcro strap was turned 180° so that the fluid inclinometer (G314S Plasty, Milano, Italy) could be fixed above the occipital bone. At least 5-minute resting period was allowed between measurements.

### Neck Extensor Endurance Test (NEE test)

**Figure 2:**
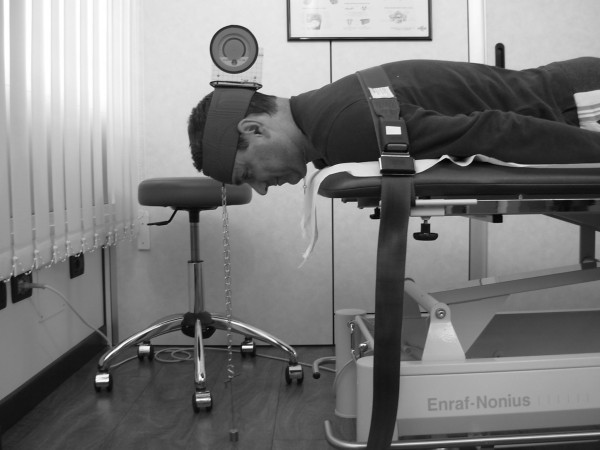
**NEE test.** The subject is lying prone with his head off the plinth, his arms by his side and his thoracic spine supported by a belt. The inclinometer above the occipital bone and the pendulum help to monitor the head position during the test.

The subject was put in a prone position with the head protruding from the plinth [7,19,30,31], and supported on a stool, arms at side and a physiotherapy belt was fastened and tightened across the T6 level in order to support the upper thoracic spine. A plumb line was fixed underneath the Velcro strap attached around the subject’s head and a small weight suspended from it (105 grams altogether), which hung to just short of the floor. Then the subject was asked to retract the chin and hold the head steady in a horizontal position while the stool was removed (Figure 2). At this point the stopwatch was started and the endurance time was measured in seconds.

The test was stopped if the subject could not maintain the head in a horizontal position (the suspended weight touched the floor), or if the subject lost more than 5° of chin tuck for over three seconds (measured with the inclinometer). The test was also discontinued if the subject wanted to stop because of fatigue or pain. After the NEE test the subject was asked to stay prone with his/her head supported for 1 minute and then to sit up.

### Statistical analysis

Data were analyzed by SPSS version 15.0 (IBM Corp., Armonk, NY, USA). The Kolgomorov-Smirnov test was used to analyze the normal distribution of the variables (p > 0.05). To compare the two groups (acute/subacute or chronic NP), an unpaired t test, a Mann–Whitney U test, or a chi-square test was performed, depending on the nature and distribution of the data. An initial analysis of variance was used to compare the groups in terms of their age and gender.

Pearson’s correlation coefficient was used to assess the bivariate associations of the variables intensity of pain (VAS), NP and disability (NPDS-I), endurance of the flexor neck muscles (NFME test) and endurance of the extensor neck muscles (NEE test). We considered this statistic, in keeping with the recommendations of Atkinson and Nevill [32], as follows: fair correlation when inferior to 0.30, moderate when ranging from 0.30 to 0.60 and good when superior to 0.60. The statistical analysis was conducted at a 95% confidence level, and p < 0.05 was considered statistically significant.

## Results

Thirty-three subjects agreed to participate in this study. Following a thorough anamnesis, history-taking, and filling out a body chart, three of these subjects were excluded from the study: two of them had shoulder pain without cervical involvement, and one subject suffered from a genetic disease affecting the face and shoulder girdle musculature (FSH Dystrophy). Thirty subjects were included, of which 18 (60%) were female and 12 (40%) were male. The mean age was 43 **±** 12 years. Sixteen subjects (53.3%) were referred from physical therapists, eight subjects (26,7%) by means of notices on the Internet boards, six subjects (20%) came with a referral from physicians.

In the 30 subjects, the performance of the NEE test was on average 246.7 ± 150 seconds (range = 48-661), whereas the performance of the NFME test was on average 44.9 ± 25.3 seconds (range = 11-112). Seven subjects in NEE test and six subjects in NFME had less than 50% of the mean score and only two subjects had more than 100% of the mean score in both tests. More than the 50% of subjects demonstrated endurance rates near the mean values in both tests. The subjects reported a mean pain VAS score of 36.67 ± 23.71 and a mean NPDS-I score of 42.95 ± 17.61.

A significant correlation was found between the results of the two endurance muscle tests (r = 0.52, p = 0.003) and a positive relationship was also found between VAS and NPDS-I (r = 0.55, p = 0.002) in the entire sample. No relevant correlation was found between the endurance tests and pain or disability (all, r < 0.26, p > 0.21)

Two included people (6.66%) complained of acute pain, 11 (36.66%) of subacute pain, and 17 (56.66%) of chronic pain. Due to the very small number of acute cases, we decided to merge acute and subacute subjects: as a consequence, our further analyses considered two different groups, which we named acute/subacute and chronic. There was no significant difference between these two groups with regard to age, sex, localization or frequency of the symptoms, referred symptoms along their arms or referred symptoms such as headache, migraine, and dizziness (Table 1). Moreover, compared with acute/subacute group, chronic NP group had no statistically significant difference in intensity of NP, although the score of NPDS-I was significantly higher in chronic group (Table 2). Endurance of the neck extensor and neck flexor muscles appeared similar in these two groups. There were no significant differences between the groups in terms of reason to stop the isometric endurance muscle tests (pain, other symptoms, tension, fear, muscular or psychological fatigue). A significant effect of gender on NFME test was revealed [F_[1,26]_ = 9.2, p < 0.001], but no effects of age on endurance test were demonstrated.

**Table 1:** Subject characteristics for each group

**Characteristic**	**Acute/subacute pain (n = 13)**	**Chronic pain (n = 17)**	**p**
Age,y, X(SD)*	45.62 (11.65)	42.59 (13.77)	0.53
Male/Female (n)***	4/9	8/9	0.36
Symptoms localization (n)^a^***	1/0/1/4/7	0/1/4/12	0.42
Frequency (n)^b^**	6/7/0	2/12/3	0.05
Upper extremity irradiation: yes/no (n)***	10/3	10/7	0.29
Other symptoms (n)^c^	2/5/3/3	4/4/1/8	0.31

Differences between groups were analyzed with a Student unpaired t test*, a Mann–Whitney U test**, or a Chi-square test*** as appropriate.

^a^The categories for the characteristic “symptoms localization” are: upper cervical spine/lower cervical spine/trapezius muscle and shoulder/two areas/three areas.

^b^The categories for the characteristic “frequency” are: sometimes/often/constant.

^c^The categories for the characteristic “other symptoms” are: no symptoms/headache/vertigo or dizziness/headache and other.

**Table 2:** Between groups differences for pain intensity, neck disability and muscle endurance tests

**Characteristic**	**Acute/subacute pain (n = 13)**	**Chronic pain (n = 17)**	**p**
VAS, X(SD)*	41.23 (24.48)	33.18 (23.23)	0.36
NPDS-I, X(SD)*	34.46 (17.64)	49.44 (15.01)	0.01
NFME test, mo (range)^a^**	44 (27–60)	30 (23.5-61.5)	0.50
NEE test, X(SD)*	261.92 (137.46)	235.12 (162.13)	0.63

Differences between groups were analyzed with a Student unpaired t test* or a Mann–Whitney U test** as appropriate.

^a^Measures with a non-Gaussian distribution are expressed as median and interquartile range (25th-75th).

In chronic NP group, VAS score and NPDS-I were significantly correlated (r = 0.696, p = 0.001). In acute/subacute NP group, intensity of pain and NPDS-I were also significantly correlated (r = 0.705, p = 0.007), and there was another significant correlation between the endurance of the flexor and extensor neck muscles (r = 0.810, p = 0.001).

Similar duration (p = 0.42) and intensity of pain (p = 0.75) and level of disability (p = 0.44) were demonstrated in males and females subgroups, in our sample. The male group lasted more time the isometric contraction in NFME test (59.33 ± 29.36 seconds) compared to female group (35.33 ± 16.98 seconds). The Mann–Whitney test showed this difference statistically significant (p = 0.035), however no other significant differences were found between these groups. In female group a positive relationship was found between VAS and NPDS-I (r = 0.55, p = 0.016), in male group the relationship was between endurance muscle tests (r = 0.781, p = 0.003).

## Discussion

Our study showed significantly higher endurance of neck extensor muscles compared to flexor ones and significant relationships between pain and disability and between NFME and NEE tests. No significant relationship between each of the endurance tests, pain and disability in subjects with neck pain were found.

Results did not significantly differ between acute/subacute, and chronic subjects, nonetheless chronic subjects appeared more disabled. Falla et al. [23] had demonstrated the lack of relationship between fatigability and duration of symptoms in chronic subjects, however a relevant less endurance in chronic subjects compared to acute/subacute ones was expected. Similar characteristics for amount and location of pain in these subgroups might explain these results, despite the higher disability of chronic subjects in the sample. Another reason may be that the chronic subjects did not have fear of movement or catastrophization [33,34].

Regarding the age of subjects, no significant relationship between age and muscle endurance was found. The analyses of gender in our sample showed a significantly higher endurance on NFME test for male subjects*.* This finding is coherent with the study of Grimmer & Trott [18], Peolsson et al. [35,36], and Kumar at al [37]. Hormonal differences between sex might have indirect effect on muscle strength production [38,39].

NEE test results differ significantly from those of Edmondston et al. [19,21] and Lee et al. [7] (Table 3). In the latter study, an extendable tape measure (approximately 20 gr) was used. Edmondston et al. [19,21] modified NEE test proposed by Lee et al. [7] added a 2 kg weight to the bottom part of the chain, in order to reduce the duration of the test.

**Table 3:** Comparison among NEE test findings of different studies

***NEE test***		
***Author***	***Max holding time (s) and standard deviation (SD) or inter-quartile range (IQR) in symptomatic subjects***	***Sample characteristics***
Parazza et al.	246.73 (SD = 150)	Group of symptomatic subjects, none in treatment at the time of the test
Edmondston et al. 2008 [19]	151.5 (SD = 71.4) 1^st^ repetition	Group of postural neck pain subjects with the addition of 2 kg weight
	149.2 (SD = 87.1) 2^nd^ repetition	
	125.0 (SD = 65.9) 3^rd^ repetition	
Edmondston et al. 2011 [21]	Median = 165 (IQR = 111–240)	Group of postural neck pain female subjects with the addition of 2 kg weight
Lee et al. 2005 [7]	350.4 (SD = 199.3)	Group of symptomatic subjects who required treatment

The NFME test was always performed first and in more than 50% of the sample it was stopped for pain or pain associated with fatigue or fear. The NFME test results are in line with the study by Edmondston et al. [19], where head and neck positioning with verbal and tactile feedback were continuously assisted. Results of this study differ from the findings obtained by Piper et al. [20], Harris et al. [26], Placzek et al. [40], and Blizzard et al. [41] (Table 4). This could be due to the differences related to the characteristics of the groups, the examiner’s methodology, or simply to physiological differences in normal populations.

**Table 4:** Comparison among NFME test findings of different studies

***NFME test***		
***Authors***	***Max holding time (s) and Standard Deviation (SD) or Inter-quartile range (IQR) in symptomatic subjects***	***Sample characteristics***
Parazza et al.	44.9 (SD = 25.8)	Group of symptomatic subjects, none in treatment at the time of the test
Blizzard et al. 2000 [41]	16.6 (SD = 4.3) 1st measurement	Group of never-injured adults from a comprehensive listing of the source population
	17.2 (SD = 3.8) 2nd measurement	
Edmonston et al. 2008 [19]	46.9 (SD = 22.7) 1st repetition	Group of postural neck pain subjects
	50.5 (SD = 25.6) 2nd repetition	
	54.1 (SD = 26.3) 3rd repetition	
Edmonston et al. 2011 [21]	Median = 38 (IQR = 14-83)	Group of postural neck pain female subjects with the addition of 2 kg weight
Harris et al. 2005 [26]	24.1 (SD = 12.8)	Group of subjects with neck pain
Piper 2009 [20]	21.83 (SD = 20.07)	Group of subjects with neck pain
Placzek et al. 1999 [40]	24.1 (SD = 17.6)	Group of subject experiencing headache

According to the findings of this study, the NFME test could be considered a useful tool in the clinical setting as an examiner could use it to quickly assess the endurance of the cervical flexors without needing any complicated or expensive equipment.

### Limitations of the study

We recognize that the sample size was small, although sufficient to determine significance and the lack of a control group. However, since this pathology is often accompanied with depression and neurodegenerative disorders, we had an important number of subjects with NP that were excluded of the study. The assessment of the subjects was carried out by a sole examiner, and gender differences were identified. The inclusion criteria considered a diagnosis of non-specific NP, regardless of the presence of cognitive or behavioral dysfunctions, especially in chronic subjects. Finally, we did not perform any intra-examiner reliability tests, so we cannot comment on the reliability of the clinical tests performed in the study.

## Conclusion

A significant correlation was found between the results of these two tests, and a positive relationship was also found between VAS and NPDS-I in subjects with neck pain. We did not find any significant differences in results between acute/subacute and chronic subjects, whereas the comparison between males and females showed high endurance for male group in NFME test. Comparison between this study and other similar ones demonstrated some variability of endurance during the NMFE or NEE tests between different samples. On the other hand, the NEE test might be less useful in a clinical situation because it is time-consuming.

## Abbreviations

CCF + CF: Cranio- Cervical Flexion + Cervical Flexion Test; DCF: Deep cervical flexors; NEE: Neck extensor endurance test; NFME: Neck flexor muscle test; NP: Neck pain; NPDS-I: Neck pain and disability scale - Italian version; SD: Standard deviation; T6: Sixth thoracic vertebra; VAS: Visual analogue scale; WAD: Whiplash associated disorders.

## Competing interests

The authors declare that they have no competing interests.

## Authors’ contribution

SP, JMT and CV provided idea for the research and created the hypothesis; EE, SP and CV planned the methods to generate the results; CV, CO’R and SP were responsible for organization and implementation; SP, CV, CO’R, and JHV wrote the manuscript; EE and SP were responsible for experiments, patient management, organization and reporting data; EE and SP were responsible for statistical analysis, evaluation, and presentation of the results; SP performed the literature search; SP, CV and CO’R were responsible for writing a substantive part of the manuscript; JMT, JHV and CV revised manuscript. All authors read and approved the final manuscript.

## References

[B1] Vos T Flaxman AD Naghavi M Lozano R Michaud C Ezzati M Years lived with disability (YLDs) for 1160 sequelae of 289 diseases and injuries 1990–2010: a systematic analysis for the Global Burden of Disease Study 2010 Lancet 2012 15 380 2163 2196 23245607 10.1016/S0140-6736(12)61729-2 PMC6350784

[B2] Mayer J Kraus T Ochsmann E Longitudinal evidence for the association between work-related physical exposures and neck and/or shoulder complaints: a systematic review Int Arch Occup Environ Health 2012 85 587 603 10.1007/s00420-011-0701-0 22038085

[B3] Carroll LJ Hogg-Johnson S Côté P van der Velde G Holm LW Carragee EJ Hurwitz EL Peloso PM Cassidy JD Guzman J Nordin M Haldeman S Course and prognostic factors for neck pain in workers: results of the bone and joint decade 2000–2010 task force on neck pain and its associated disorders Spine 2008 33 4 Suppl S93 S100 15 18204406 10.1097/BRS.0b013e31816445d4

[B4] Walton DM Macdermid JC Giorgianni AA Mascarenhas JC West SC Zammit CA Risk factors for persistent problems following acute whiplash injury: update of a systematic review and meta-analysis J Orthop Sports Phys Ther 2013 43 2 31 43 10.2519/jospt.2013.4507 23322093

[B5] Saavedra-Hernández M Castro-Sánchez AM Cuesta-Vargas AI Cleland JA Fernández-de-las-Peñas C Arroyo-Morales M The contribution of previous episodes of pain, pain intensity, physical impairment, and pain-related fear to disability in patients with chronic mechanical neck pain Am J Phys Med Rehabil 2012 91 1070 1076 10.1097/PHM.0b013e31827449a5 23159953

[B6] Jull G Whiplash, Headache, and Neck Pain: Research-Based Directions for Physical Therapies 2008 Edinburgh; New York: Churchill Livingstone/Elsevier

[B7] Lee H Nicholson LL Adams RD Neck muscle endurance, self-report, and range of motion data from subjects with treated and untreated neck pain J Manip Physiol Ther 2005 28 25 32 10.1016/j.jmpt.2004.12.005 15726032

[B8] Elliott J Jull G Noteboom JT Darnell R Galloway G Gibbon WW Fatty infiltration in the cervical extensor muscles in persistent whiplash-associated disorders: a magnetic resonance imaging analysis Spine 2006 31 E847 E855 10.1097/01.brs.0000240841.07050.34 17047533

[B9] Elliott J Jull G Noteboom JT Galloway G MRI study of the cross-sectional area for the cervical extensor musculature in patients with persistent whiplash associated disorders (WAD) Man Ther 2008 13 258 265 10.1016/j.math.2007.01.012 17383216

[B10] Uhlig Y Weber BR Grob D Muntener M Fiber composition and fiber transformations in neck muscles of patients with dysfunction of the cervical spine J Orthop Res 1995 13 240 249 10.1002/jor.1100130212 7722761

[B11] Elliott J Sterling M Noteboom JT Darnell R Galloway G Jull G Fatty infiltrate in the cervical extensor is not a feature of chronic, insidious-onset neck pain Clin Radiol 2008 63 681 687 10.1016/j.crad.2007.11.011 18455560

[B12] Kristjansson E Reliability of ultrasonography for the cervical multifidus muscle in asymptomatic and symptomatic subjects Man Ther 2004 9 83 88 10.1016/S1356-689X(03)00059-6 15040967

[B13] Falla D Jull G Hodges PW Patients with neck pain demonstrate reduced activation of the deep neck flexor muscles during performance of the craniocervical flexion test Spine 2004 29 2108 2114 10.1097/01.brs.0000141170.89317.0e 15454700

[B14] Falla D Jull G Hodges PW Feedforward activity of the cervical flexor muscles during voluntary arm movements is delayed in chronic neck pain Exp Brain Res 2004 157 43 48 10.1007/s00221-003-1814-9 14762639

[B15] Falla D Bilenkij G Jull G Patients with chronic neck pain demonstrate altered patterns of muscle activation during performance of a functional upper limb task Spine 2004 29 1436 1440 10.1097/01.BRS.0000128759.02487.BF 15223935

[B16] O’Leary S Falla D Elliott JM Jull G Muscle dysfunction in cervical spine pain: implications for assessment and management J Orthop Sports Phys Ther 2009 39 324 333 10.2519/jospt.2009.2872 19411767

[B17] Grimmer K Measuring the endurance capacity of the cervical short flexor muscle group Aus J Physiother 1994 40 251 254 10.1016/S0004-9514(14)60461-X 25025606

[B18] Grimmer K Trott P The association between cervical excursion angles and cervical short flexor muscle endurance Aus J Physiother 1998 44 201 207 10.1016/S0004-9514(14)60380-9 11676734

[B19] Edmondston SJ Wallumrød ME MacLéid F Kvamme LS Joebges S Brabham GC Reliability of isometric muscle endurance tests in subjects with postural neck pain J Manip Physiol Ther 2008 31 348 354 10.1016/j.jmpt.2008.04.010 18558277

[B20] Piper A Comparison of endurance capacity of deep cervical flexors between healthy and subjects with cervical pain [German: Vergleich der ausdauerleistungsfähigkeit der vorwiegend tiefen HWS-flexoren zwischen gesunden und probanden mit HWS-schmerz] Manuelle Therapie 2009 13 202 211

[B21] Edmondston SJ Björnsdóttir G Pálsson T Solgard H Ussing K Allison G Endurance and fatigue characteristics of the neck flexor and extensor muscles during isometric tests in patients with postural neck pain Man Ther 2011 16 332 338 10.1016/j.math.2010.12.005 21256071

[B22] Cagnie B Dickx N Peeters I Tuytens J Achten E Cambier D Dannells L The use of functional MRI to evaluate cervical flexor activity during different cervical flexion exercises J Appl Physiol 2008 104 230 235 17991788 10.1152/japplphysiol.00918.2007

[B23] Falla D Rainoldi A Jull G Stavrou G Tsao H Lack of correlation between fatigability of the sternocleidomastoid and anterior scalene muscles and duration of symptoms in chronic neck pain patients Clin Neurophysiol 2004 3–4 159 165 10.1016/j.neucli.2004.04.004 15501686

[B24] Monticone M Baiardi P Nido N Righini C Tomba A Giovanazzi E Development of the Italian version of the neck pain and disability scale, NPDS-I: cross-cultural adaptation, reliability, and validity Spine 2008 33 429 434 10.1097/BRS.0b013e318175c2b0 18520930

[B25] Wheeler AH Goolkasian P Baird MA Darden BV Development of the neck pain and disability scale Spine 1999 24 1290 1294 10.1097/00007632-199907010-00004 10404569

[B26] Harris KD Heer DM Roy TC Santos DM Whitman JM Wainner RS Reliability of a measurement of neck flexor muscle endurance Phys Ther 2005 12 1349 1355 16305273

[B27] Olson L Millar A Dunker J Hicks J Glanz D Reliability of a clinical test for deep cervical flexor endurance J Manip Physiol Ther 2006 29 134 138 10.1016/j.jmpt.2005.12.009 16461172

[B28] Caigne B Cools A De Loose V Cambier D Danneels L Differences in isometric neck muscle strength between healthy control and women with chronic neck pain: the use of a reliable measurement Arch Phys Med Rehabil 2007 88 1441 1445 10.1016/j.apmr.2007.06.776 17964885

[B29] Childs JD Cleland JA Elliott JM Teyhen DS Wainner RS Whitman JM Sopky BJ Godges JJ Flynn TW Association APT Neck pain: clinical practice guidelines linked to the international classification of functioning, disability, and health from the orthopaedic section of the American Physical Therapy Association J Orthop Sports Phys Ther 2008 38 A1 A34 18758050 10.2519/jospt.2008.0303

[B30] Biering-Sorensen F A one-year prospective study of low back trouble in a general population. The prognostic value of low back history and physical measurement Spine 1984 31 362 375 6239755

[B31] Ljungquist T Fransson B Harms-Ringdahl K Björnham Å Nygren Å A physiotherapy test package for assessing back or neck dysfunction – discriminative ability for patients versus healthy control subjects Physiother Res Int 1999 4 123 140 10.1002/pri.158 10444762

[B32] Atkinson G Nevill A Comment on use of concordance correlation to assess the agreement between two variables Biometrics 1997 52 775 777

[B33] Monticone M Baiardi P Bonetti F Ferrari S Foti C Pillastrini P Rocca B Vanti C Zanoli G The Italian Version of the Fear Avoidance Belief Questionnaire, FABQ-I. Cross-cultural adaptation, factor analysis, reliability, validity, and sensitivity to change Spine 2012 37 E374 E380 10.1097/BRS.0b013e31822ff5a7 22422439

[B34] Monticone M Baiardi P Ferrari S Foti C Mugnai R Pillastrini P Rocca B Vanti C Development of the Italian version of the Pain Catastrophising Scale (PCS-I): cross-cultural adaptation, factor analysis, reliability, validity and sensitivity to change Qual Life Res 2012 21 1045 1050 10.1007/s11136-011-0007-4 21912846

[B35] Peolsson A Almkvist C Dahlberg C Lindqvist S Pettersson S Age- and sex-specific reference values of a test of neck muscle endurance J Manip Physiol Ther 2007 30 171 177 10.1016/j.jmpt.2007.01.008 17416270

[B36] Peolsson A Oberg B Hedlund R Intra- and inter-tester reliability and reference values for isometric neck strength Physiother Res Int 2001 6 15 26 10.1002/pri.210 11379253

[B37] Kumar S Narayan Y Amell T Cervical strength of young adults in sagittal, coronal, and intermediate planes Clin Biomech 2001 16 380 388 10.1016/S0268-0033(01)00023-7 11390044

[B38] Strimpakos N The assessment of the cervical spine. Part 2: strength and endurance/fatigue J Bodyw Mov Ther 2011 15 417 430 10.1016/j.jbmt.2010.10.001 21943615

[B39] Mc Ardle WD Katch FI Katch VL Exercise Physiology, Energy, Nutrition, and Human Performance 1991 London: William and Wilkins

[B40] Placzek JD Pagett BT Roubal PJ Jones BA McMichael HG Rozanski EA Gianotto KL The influence of the cervical spine on chronic headache in women: a pilot study J Man Manip Ther 1999 7 33 39 10.1179/106698199790811924

[B41] Blizzard L Grimmer KA Dwyer T Validity of a measure of the frequency of headaches with overt neck involvement, and reliability of measurement of cervical spine anthropometric and muscle performance factors Arch Phys Med Rehabil 2000 81 1204 1210 10.1053/apmr.2000.7168 10987163

